# Image Noise Removal in Ultrasound Breast Images Based on Hybrid Deep Learning Technique

**DOI:** 10.3390/s23031167

**Published:** 2023-01-19

**Authors:** Baiju Babu Vimala, Saravanan Srinivasan, Sandeep Kumar Mathivanan, Venkatesan Muthukumaran, Jyothi Chinna Babu, Norbert Herencsar, Lucia Vilcekova

**Affiliations:** 1 School of Computer Science and Engineering, Vellore Institute of Technology, Vellore 632014, India; 2 Department of Computer Science and Engineering, Vel Tech Rangarajan Dr. Sagunthala R&D Institute of Science and Technology, Chennai 600062, India; 3 School of Information Technology and Engineering, Vellore Institute of Technology, Vellore 632014, India; 4 Department of Mathematics, College of Engineering and Technology, SRM Institute of Science and Technology, Kattankulathur 603203, India; 5 Department of Electronics and Communications Engineering, Annamacharya Institute of Technology and Sciences, Rajampet 516126, India; 6 Department of Telecommunications, Faculty of Electrical and Communication Engineering, Brno University of Technology, Technicka 12, 616 00 Brno, Czech Republic; 7 Faculty of Management, Comenius University Bratislava, Odbojarov 10, 820 05 Bratislava, Slovakia

**Keywords:** local speckle noise destruction, hybrid deep learning technique, logical-pool recurrent neural network, signal-to-noise ratio, spatial high-pass filter, glandular ultrasound image

## Abstract

Rapid improvements in ultrasound imaging technology have made it much more useful for screening and diagnosing breast problems. Local-speckle-noise destruction in ultrasound breast images may impair image quality and impact observation and diagnosis. It is crucial to remove localized noise from images. In the article, we have used the hybrid deep learning technique to remove local speckle noise from breast ultrasound images. The contrast of ultrasound breast images was first improved using logarithmic and exponential transforms, and then guided filter algorithms were used to enhance the details of the glandular ultrasound breast images. In order to finish the pre-processing of ultrasound breast images and enhance image clarity, spatial high-pass filtering algorithms were used to remove the extreme sharpening. In order to remove local speckle noise without sacrificing the image edges, edge-sensitive terms were eventually added to the Logical-Pool Recurrent Neural Network (LPRNN). The mean square error and false recognition rate both fell below 1.1% at the hundredth training iteration, showing that the LPRNN had been properly trained. Ultrasound images that have had local speckle noise destroyed had signal-to-noise ratios (SNRs) greater than 65 dB, peak SNR ratios larger than 70 dB, edge preservation index values greater than the experimental threshold of 0.48, and quick destruction times. The time required to destroy local speckle noise is low, edge information is preserved, and image features are brought into sharp focus.

## 1. Introduction

The process of noise removal from an image has been studied for decades as academics attempt to tackle this “classical challenge”. Filters were once used by scientists in order to lessen the visual disturbances in photographs. In the past, they were effective up to a certain degree of noise in an image. However, using such filters would cause the image to blur. In addition, if the image is overly noisy, the final product will be so fuzzy that important information will be lost [1]. Breast illness is on the rise, and breast hyperplasia is the most prevalent breast condition, while malignancy is the most frequent female malignancy. Early identification and efficient treatment boost the clinical outcome of breast illness, according to this research [2]. Ultrasound imaging technology is now used to diagnose and treat breast cancer. Due to its low cost and good performance, ultrasonic imaging is used to identify breast cancer. Breast ultrasound imaging is an excellent tool for checking breast health. Ultrasound scattering causes speckle noises. Speckle noise lowers image resolution, alters how the clinician interprets the ultrasound image, and prevents accurate health monitoring and assessment [3]. Consequently, the significance of the research into reducing the speed of the speckles in ultrasound imaging of the breast cannot be overstated. Complex machine learning techniques such as three-dimensional deep learning efficiently handle image data. As shown, the proposed deep learning classification method successfully identified data based on both local and global features. The use of deep learning for the interpretation and investigation of breast ultrasound images was more effective and used less computing power than traditional approaches. Using a Deep Convolutional Neural Network (DCNN) [4], we developed a method for automatically classifying lesions in ultrasound images of the thyroid and breast. With the same architecture and transfer learning in mind, we proposed a general DCNN structure parameter setting and evaluated this overarching method using real-world ultrasound images. The authors conduct a comprehensive comparison of deep image noise-removal algorithms. We begin by separating the DCNN into four categories: additive white noise images, genuine noisy images, blind noise removal, and hybrid noisy images. We then compare and contrast the goals and underlying ideas of the various deep learning approaches. Next, we conduct a quantitative and qualitative comparison of the state-of-the-art approaches using publicly available datasets for noise removal. Finally, we suggest several obstacles and avenues for further study [5]. Facial recognition algorithms function best in laboratory conditions where lighting, expression, and position may all be carefully manipulated. There have been studies using infrared (IR) and three-dimensional (3D) images of faces for facial recognition. It has also been shown in studies that fusing various facial modalities yields better results than using a single one. Noise removal of the image is considered a common problem in the image-processing domain and computer-aided vision, where the objective is to approximate the original image by reducing noise. Image noise is frequently unavoidable due to intrinsic (sensor) and extrinsic (environment) factors. In several applications, including image restoration, eye tracking, image registration, segmentation techniques, and image classification, recovering the actual image content is critical for good performance. While various methods have been developed for image noise removal, image noise reduction remains difficult, particularly when images are collected under poor conditions with excessive noise [6].

The proposed technique suppresses local speckle noise in ultrasound breast images, retains edge information, and makes image features apparent, laying the groundwork for intelligent ultrasound image processing and application. This study will focus on improving the versatility of the investigation of different types of noise and other features of ultrasound, MRI, and CT image characteristics as well.

## 2. Literature Review

Deep learning is used for noise removal in the image. Deep learning algorithms for image noise removal vary greatly. Deep learning-based discriminative learning can handle Gaussian noise. Deep learning-based optimization methods estimate actual noise. Few studies have summarized deep learning image noise-removal algorithms [7]. Noise removal is crucial for medical imaging analysis, diagnosis, and therapy. Deep learning-based image noise-removal algorithms are effective but restricted by sample. We develop a deep feed forward noise-removal CNN for medical image noise removal using a modest sample set to demonstrate a new method for solving basic eyesight issues [8]. We propose a different training program that effectively adapts, initially conceived for unsupervised learning, to the activities of image noise removal and blind inpainting. This is accomplished with the help of encoding and deep networks that have been pre-trained with a noise-removal auto-encoder. For the image noise removal challenge, our solution performs just as well as the popular K-singular value decomposition (KSVD) sparse coding method [9]. In this technique for image noise removal, contrast is adaptively increased. It is not always feasible to get high-quality photos of whatever is trying to be captured. This is due to the fact that they were shot under a wide range of lighting conditions. Because of this, the perceived quality of the obtained photographs is low. As a result, it is crucial to boost the quality of photographs while keeping edge detail intact as much as possible [10]. The purpose of this study is to offer a thorough overview of the most up-to-date developments in deep neural network-based image noise-removal methods. To achieve this, we first provide a detailed explanation of the problem statement surrounding image noise removal, before moving on to the specifics of the datasets used as standards and the metrics often used during objective evaluations [11]. To comprehend the area and advancement of deep learning in noise removal, research scholars, academics, and industry experts should evaluate numerous methodologies. The research presented here presents three distinct noise-canceling frameworks: the wavelet, the pulse-coupled, and the CNN [12]. The noise removal uses fast CNN architecture. The spatial-wise attention residual network (SARN) method helps train bigger, better datasets. SARN CNN preserves details and image edges during reconstruction [13]. Image restoration removes noise and creates a copy as similar to the original as is feasible. This study focuses on feed-forward noise removal. Using a convolutional neural network (CNN), we can deblock Joint Photographic Experts Group (JPEG) images, improve resolution using super-resolution, and remove blinding Gaussian noise. Using batch normalization and residual training boosts noise-removal performance and reduces operation time [14]. Using the raw information available in k-space, we compare two unsupervised methods for noise removal in magnetic resonance imaging (MRI) images in the complex image space. Both approaches are based on Stein’s unbiased risk estimator, although the latter is more restrictive due to its use of a blind-spot network. Both approaches are put through their paces on two datasets: one with genuine MRI scans of knees and the other with simulated scans of the brain. These datasets will be utilized for noise removal since they include information on the intricate image space [15]. In the presented approach, a stretched convolutional neural network is combined with pre- and post-processing methods to enlarge the receptive field. As a result, big distance pixels in source images will help enrich the image features of the learned model, which effectively denoises the images [16]. The author proposes a lightweight, effective neural network-based raw image denoiser that works well on popular mobile devices and yields great-quality noise-removal results. The two main takeaways from our research are: (i) a simpler network trained on synthetic sensor-specific data may outperform bigger networks trained on general data; and (ii) a unique k-Sigma Transform can reduce the huge noise level variance under a varied international organization for standardization (ISO) settings, enabling a tiny network to effectively manage a broad range of noise levels [17]. The author suggests a new deep network design for non-local image noise removal, which may be used for both grayscale and colour images. The variational approaches that make use of the non-local self-similarity feature of natural images have inspired the general architecture of the proposed network. Using this idea as a foundation, we develop deep networks capable of non-local processing that also greatly benefit from discriminative training [18]. Due to the complex nature of data collection, developing an unsupervised speckle-reduction solution for practical purposes is a difficult challenge. In practice, the distortion distribution is often too complicated for the simple additive white Gaussian hypothesis to hold, which severely compromises the effectiveness of Gaussian denoisers [19]. Classifying HIs improves clinicians' abilities to diagnose ailments and treat patients. Due to their ability to automatically extract characteristics, deep learning algorithms have found widespread use in a number of different sectors, most notably medical imaging [20].

## 3. Proposed Framework Methodologies

### 3.1. Dataset Availability

INbreast and CBIS-DDSM (Curated Breast Imaging Subset-Digital Database for Screening Mammography) datasets were used to collect the experimental data. Dataset from INbreast: There were 120 instances (412 photos) in INbreast, 91 of them originated with females who had both breasts (four images each), whereas the remaining 30 were from people who had had a mastectomy (2 images each). There were a number of different inflammatory lesions that caused deformities. The expert also sent us the detailed outlines in extensible markup language (XML) format. Data from the CBIS-DDSM: The breast data set, which is one of the most popular and extensive data collections, is split into four different directories. The breast dataset is a well-known and sizable data collection that has been subdivided into benign without call-backs, benign, malignancies, and normal. Each folder contains several instances, each representing a sample of a certain kind of breast examination. Using the proposed approach in this paper, we rapidly destroy local speckle noise in a sample of 1000 ultrasound breast images drawn from the aforementioned two datasets, where 800 images are utilized to prepare a logical-pool recurrent neural network, and the remaining 200 images are utilized as test samples during analysis.

### 3.2. Effective Destruction of Local Speckle Noise in Breast Images

**Figure 1:**
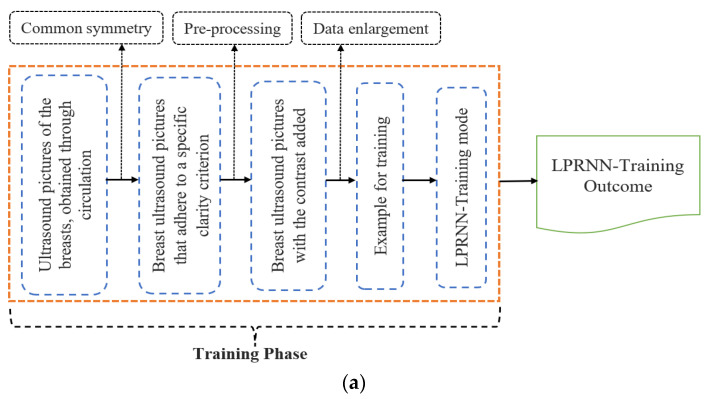

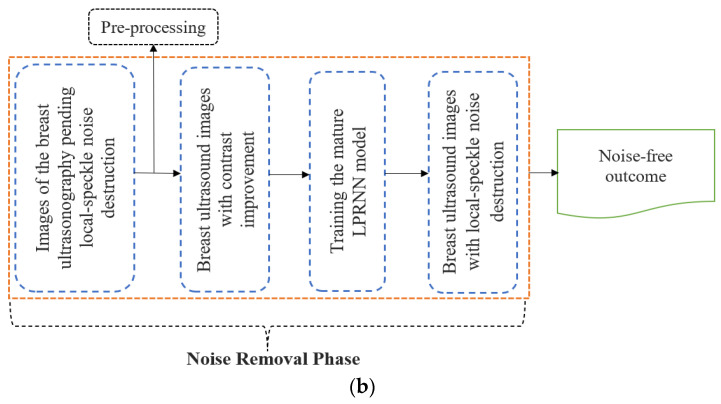
Local speckle denoising method for breast ultrasound images.

Most methods for reducing the amount of noise in ultrasound images take the form of an additive background noise model. The ultrasound breast local speckle noise reduction while protecting the confidentiality of data at the edge was achieved by using a Logical-Pool Recurrent Neural Network (LPRNN) for the local speckle destruction method. Figure 1 shows this to be the case. The method of local speckle noise removal from an ultrasound breast image consists mostly of three steps: pre-processing, training, and denoising. Initially, the obtained ultrasound breast images are aligned, the standardized and clear ultrasound images are located, and the contrast increase processing is finished. After the data has been processed, data expansion processing is carried out in order to create training samples, and the LPRNN is then obtained by training on these samples. Contrast enhancement processing utilizing the trained LPRNN is followed by speckle denoising of the ultrasound breast images. Figure 1 depicts the local speckle denoising method for breast ultrasound images, and it has two different phases: (a) training phase; (b) noise-free phase. Segmenting digital images is crucial in many disciplines.

Image segmentation separates objects from the backdrop. Object detection requires image segmentation. Noisy images are more problematic. Noises such as salt-and-pepper, Gaussian, Poisson, and speckle damage most digital photos. Speckle noise impacts pixels in a grayscale image and is common in low-luminance imaging such as specific absorption rate (SAR) and MRI. Image enhancement reduces speckle noise before object identification, segmentation techniques, edge detection, etc. 

### 3.3. Breast Image Pre-Processing and Image Enhancement

It is very crucial to do pre-processing on breast ultrasound images before attempting to decrease ultrasound image local speckle noise. The algorithm for image-guided filtering is used in the operation of pre-processing ultrasound breast images. This operation is broken down into three distinct parts. The image contrast of the breast imaging that was supplied is processed in the first phase of the procedure. The algorithm of image-guided filtration is what is used in the second stage to accomplish the desired augmentation of the ultrasound image’s level of detail. In the third phase, the high-pass filtering for spatial analysis is applied to the ultrasound breast images in order to reduce the excessive sharpening that was introduced in the previous step. Ultrasound imaging of the breast may be drastically improved by adjusting the grayscale setting. Employing logarithmic and exponential transformations throughout a variety of grayscale mean M from [0, 260], this research enhances the contrast of grayscale values in breast ultrasound images as input. When applied to an input breast ultrasound image f∈(i, j), the logarithmic transform may be used to increase contrast by enlarging the lower grey value interval and compressing the upper grey value interval. The logarithmic equation can be expressed as:

(1) $$h\left(i,j\right)=\frac{a.L\left(f\in \left(i,j\right)\right)}{b}+c$$

Here, *c* and *a* illustrate the exponential and log co-efficient transformation; correspondingly, *L(.)* is considered as the logarithmic function, and *b* is the constant with the (0,1) interval value. In this research, we employed exponential transformation to handle images with too high brightness, avoiding the whitening and inadequate compensating issues that arise when using log transformation. The transformation equation of the exponential can be expressed as:

(2) $$h\left(i,j\right)=a^{b(f\in \left(i,j\right)}-a^{c}-1$$

Here, *h(i,j)* is the outcome of the image after the transformation. The combined information, along with transformation, then the equation can be written as:

(3) $$\{\begin{array}{cc} h\left(i,\;j\right)=\frac{a.L\left(f\in \left(i,j\right)\right)}{b}+c & 0\leq N<100\\ h\left(i,\;j\right)=f\in \left(i,j\right) & 100\leq N<180\\ h\left(i,j\right)=a^{b(f\in \left(i,j\right)}-a^{c}-1 & 180\leq M<260 \end{array}$$

From Equation (3), the grayscale value of the image can be modified in order to set a value after the transformation is carried out. It provides an enhanced image with respect to its contrast and offers good guide image acquisition. Linear transformation filtering is used as a guide in the image-filtering process. Therefore, a weighted mean is utilized to depict the filtered outcome *n_i_* of the *i*th pixel point of the ultrasound breast image to be local-speckle-noise destructed with contrast enhancement finished *M*, the guided-image *I_m_*, and the outcome improved ultrasound breast image *N*. The computation equation can be written as:

(4) $$n_{i}=\sum_{j}m_{j}Z_{ij}\left(I_{m}\right)$$

Here, *m_j_* indicates the *j^th^* weighted value of the average vector, $Z_{ij}\left(I_{m}\right)$ is considered as the function of the kernel filter. Then, the computational equation is written as:

(5) $$Z_{ij}\left(I_{m}\right)=\frac{\sum_{r:\left(i,\;j\right)\epsilon v_{r}}(\frac{\left(I_{mi}-\mu_{r}\right)\left(I_{mj}-\mu_{r}\right)}{\vartheta_{r}^{2}+\epsilon }}{{\left|v\right|}^{2}}$$

Here, the operation takes the image *I_m,_* and the kernel value is $Z_{ij}\left(I_{m}\right)$ and it is free of correlation along with the ultrasound breast image *M,* which is local-speckle-noise destructed. However, $v_{r}$ is referred to as the kernel window, then the pixel count in the window is denoted as |v|, then the $\mu_{r}$ and $\vartheta_{r}^{2}$ are the mean and variance, respectively, the ϵ is referred to as a smoothing operator. The guided filtering of the image for breast (ultrasound) information improvement is written as:

(6) $$I_{m}^{\prime }=n+FI_{m}-Fn$$

Here, *F* is the parameter of improved degree correction. 

### 3.4. High-Pass Ultrasound Image Spatial Filtering for Breast Images

In order to accomplish the aim of reducing the factors in a particular space, the spatial filter modifies the distribution frequency of the image, which ultimately results in an increase in the image contrast. In order to make the image clearer, the high-pass filter is used to limit the images that have a low frequency throughout the locally efficient processing of the input image. This successfully eliminates the local over-sharpening that would have been induced by the image-guided filtration function. The $H_{p}\left(u,v\right)$ is a template of a high-pass filter, and it can be expressed as:

(7) $$H_{p}\left(u,v\right)=\frac{1}{7}\left(\begin{array}{ccc} -2 & -1 & -2\\ -1 & 18 & -1\\ -2 & -1 & -2 \end{array}\right)$$

Here, the ultrasound breast image output is $b_{ui}\left(a,b\right)$ after the image guided-filtering improvement is written as:

(8) $$b_{ui}\left(a,b\right)=\sum_{u=0}^{r}f\left(a-u,\;b-v\right)\sum_{u=0}^{r}H_{p}\left(u,v\right)$$

The *u* and *v* indicate the various parameter templates of a high-pass filter, and *a* and *b* illustrate the various image parameters of ultrasound breast images, and *r* is the highest value of the template parameter. However, Equations (6) and (8) will be combined in order to obtain the ultrasound breast image spatial high-pass filter, then $I_{m}^{\prime \prime }\left(a,b\right)$ is written as:

(9) $$I_{m}^{\prime \prime }\left(a,b\right)=\sum_{u=0}^{r}I_{m}^{\prime }\left(a-u,\;b-v\right)\sum_{u=0}^{r}H_{p}\left(u,v\right)$$

The pre-processing steps of high-pass ultrasound image spatial filtering for breast images can be written as:

*Step 1: To improve the process of acquiring the bootstrap image, we first determine a grayscale value of N, of the source ultrasound breast image f, and then use Equation (3) to identify the associated breast ultrasound image h that best fits the categorization.*
*Step 2: Having completed Step 1, the ultrasound breast image is utilized as the reference image I’_m_ of the guide-filtration algorithm.*
*Step 3: I’’ is the ultrasound breast outcome, and it is achieved by improving the edge data of I’ along with a high-pass filter in order to enlarge the edge data retention.*

### 3.5. Logical-Pool Recurrent Neural Network—Local Speckle Noise Destruction

**Figure 2:**
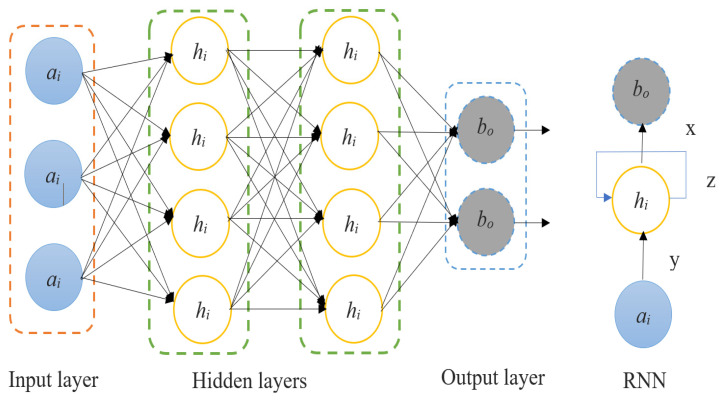
Recurrent neural network.

To address the issue of local speckle noise destruction in ultrasound breast images as an image and image mapping problem, we introduce a logical-pool recurrent neural network and implement a training model between endpoints on training samples composed of pre-processed, finished breast images. The ultrasound images may have their local speckle noise reduced by using a logical-pool recurrent neural network; however, this comes at the expense of edge blurring and some data loss. Using the groundwork laid by the introduction of logical-pool recurrent neural networks, the proposed algorithm explicitly targets edge information loss as the objective of the modification function, thereby decreasing the likelihood of edge information loss in ultrasound breast images during local speckle noise destruction. The fundamental structure of a recurrent neural network is shown in Figure 2. Feeding the ultrasound images of the breast into a logical-pool recurrent neural network model yielded results with and without the local speckle noise reduction. The layers of a logical-pool recurrent neural network model include an input layer, a convolution layer, a pooling layer, a fully connected layer, and an output layer.

**Figure 3:**
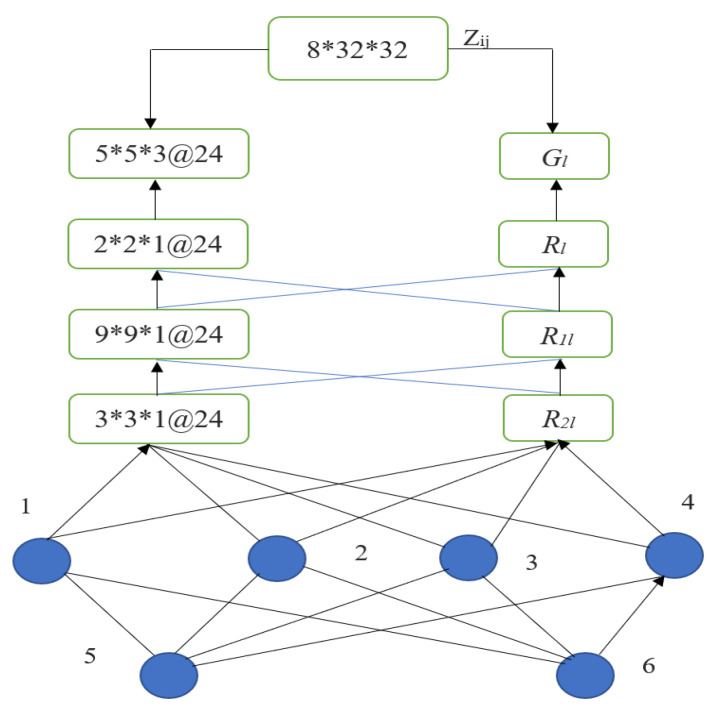
Logical-pool support RNN.

Figure 3 depicts the convolutional nodes (1,2,3,4,5 and 6) all these nodes are performing a fully connected network and it is a detailed design of a logical-pool recurrent neural network model. Layers Zij (input), Gl (convolution), Rl (pooling), R1 (complete connection), and R2 (output) are shown in Figure 3, with sizes of 8 × 32 × 32, 5 × 5 × 3, 2 × 2 × 1, 9 × 9 × 1, and 3 × 3 × 1, respectively. When the entirely connected layer is placed before the layer outcome, the convolution layer and pooling layer are arranged in counter-clockwise order, and the data flowing through and out of the convolution and pooling layers are neural network feature bodies. To select the stacked high-level features, it is necessary to first determine the logical-pool recurrent convolution kernels of all the convolution layers, then create a new feature space by combining the recurrent convolution kernels of different convolution layers, and finally activate the nonlinear function by improving the bias term. The pooling layer is the most crucial aspect of a logical-pool recurrent neural network since it may decrease the number of features without altering the local information of the features. Configuring the neural network layer as *l_n_,* and *V* is the pooling layer, and the neural network input layer is referred to and written as:

(10) $$V=\left[p_{1}^{l_{n}},p_{2}^{l_{n}},\;p_{3}^{l_{n}},\;p_{4}^{l_{n}},p_{5}^{l_{n}}\ldots p_{r}^{l_{n}}\right]\in A^{X*Y*Z*K}$$

In order to provide an output, the max pooling layer must first figure out the highest number of the cube of the number of features.

(11) $$V^{1}\in A^{X^{\prime }*Y^{\prime }*Z^{\prime }*K^{\prime }}$$

Before feature extraction, the deep feature size is (*X, Y, Z*), but after feature extraction, the deep feature size is $\left(X^{\prime },Y^{\prime },Z^{\prime }\right)$ and the *K* is referred to as the count of spaces of features. All neurons in a fully linked layer communicate with neurons in the layer above. Breast ultrasound images following the preceding pre-processing are used to train a logical-pool recurrent neural network-based image local speckle noise destruction framework, with the resulting images being the model’s output layer’s local-speckle-noise-free output.

#### Local Speckle Noise Destruction Algorithm 

**Figure 4:**
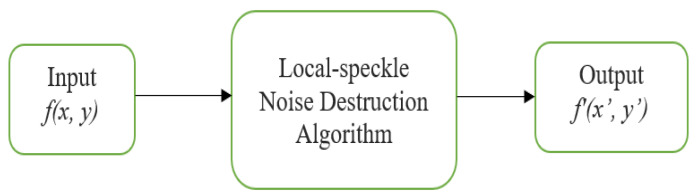
Algorithm 1 for noise removal of the local speckle noise.

Here are the operations included in the recurrent deep learning-based method that eliminates rapid local speckle noise in ultrasound breast images. Here, the input original ultrasound breast image *f(x, y)* is pre-processed, and a logical-pool recurrent neural network architecture is constructed, and the outcome is ultrasound breast images after the local speckle noise destruction, shown in Figure 4.
**Algorithm 1.** Noise removal of the local speckle noise.1:Begin2:Logarithmic and computational transforms are used to improve the differentiation of the input ultrasound breast images; the algorithm (guided filter) is used to improve the details of the glandular ultrasound images; and the spatial high-pass filtering algorithm is used to denoise the over-sharpening of the ultrasound breast images, all based on their grayscale values3:The pre-processed ultrasound breast images are fed into a local-speckle-noise destruction model of a logical-pool recurrent neural network4:Ultrasound breast images are susceptible to losing image edge information during the local speckle noise reduction procedure. If we want to preserve the edge information after local speckle noise removal is applied, we will need to understand how that information is lost during processing. The meaning of “edge information loss”.
(12)$${\mathrm{Loss}}_{\mathrm{Edge}}\left(P\right)=\log\frac{I_{m}^{\text{″}}\left(a,b\right)}{\sum_{i,j}\left|b_{i+1,j}-b_{i,j}\right|}$$5:In order to construct ultrasound image gradients, we first analyze the aforementioned stages and then use edge loss pairs to compare the edges of canonical clear images of ultrasound breast images. The unique anatomy of the breast emphasizes the significance of the gradients in the vertical plane. That is why we first use contrast in the vertical direction to depict breast ultrasound images. Integrating edge loss ${\mathrm{Loss}}_{\mathrm{Edge}}\left(P\right)$ and L1 distance with a recurrent neural network yields the following objective function:
(13)$P^{*},{\;C}^{*}=\arg\;\underset{P}{\min}\underset{C}{\max}{\;\mathrm{Loss}}_{\mathrm{HRNN}}\left(P,C\right)+{b\;\mathrm{Loss}}_{{\mathrm{Loss}}_{1}}\left(P\right)+{\beta \mathrm{Loss}}_{\mathrm{Edge}}\left(P\right)$6:Enhance the loss function to optimize the edge-specific improvement feature of the ultrasound images during training with the logical-pool recurrent neural network. The resulting model will be more responsive in edge local speckle noise destruction in ultrasound images, enhancing its effect on ultrasound breast images7:While noise removal reduces the local speckle noise of ultrasound breast images, the edge information is preserved by the action of the advantage term in the logical-pool recurrent neural network as described above8:End

### 3.6. Performance Metric Evaluation Standards

When it comes to reducing local speckle noise in the detection of ultrasound breast images, the training impact of a logical-pool recurrent neural network is greater when both the mean square error (MSE) and the false identification rate are lower. Different ultrasound breast images have varying levels of local speckle noise destruction depending on how close both the PSNR, and SNR of the denoised images, are to the respective threshold levels of 55 and 70 dB. To determine the PSNR, one does the following:

(14) $$\mathrm{Peak}\;\mathrm{signal}\;\mathrm{to}\;\mathrm{noise}\;\mathrm{ratio}=10\log_{10}\left(\frac{{\mathrm{maximum}}_{I}^{2}}{\mathrm{mean}\;\mathrm{square}\;\mathrm{error}}\right)$$

Here, ${\mathrm{maximum}}_{I}^{2}$ is the highest value color image coordinates, and the mean square error is the deviation function of the image. Results of local speckle noise removal from ultrasound breast images, as measured by the border protection index (BPI): Evaluation of the proposed algorithm’s calculation of local speckle noise destruction in ultrasonic images requires consideration of not just the signal-to-noise ratio and peak-to-average ratio, but also the degree to which image boundaries are preserved after noise-removal. When evaluating edge retention performance, a higher EPI value indicates a more robust BPI. Specifically, this is the equation used to calculate EPI from experimental data:

(15) $$Border\;protection\;index=\frac{\sum_{i=0}^{n}(\nabla a-\bar{\nabla }a)}{\sqrt{\sum_{i=0}^{n}{\left(\nabla a-\bar{\nabla }a\right)}^{2}}}-\frac{\sum_{i=0}^{n}(\nabla b-\bar{\nabla }b)}{\sqrt{\sum_{i=0}^{n}{\left(\nabla b-\bar{\nabla }b\right)}^{2}}}$$

Here, *a* is the image before the ultrasound, *b* is the image after the ultrasound has removed the local speckle noise, and ∇ is referred to as the Laplace-operator. The effectiveness of local speckle noise destruction in ultrasound breast images is proportional to the improvement in image quality achieved by noise removal.

(16) $$T_{des}=\sum_{k=1}^{m}t_{des}^{k}$$

The above formula is used to calculate the time till destruction. The effectiveness of local speckle noise removal in ultrasound breast images is proportional to the amount of time spent on image noise removal after the use of various approaches.

## 4. Experimental Results 

**Figure 5:**
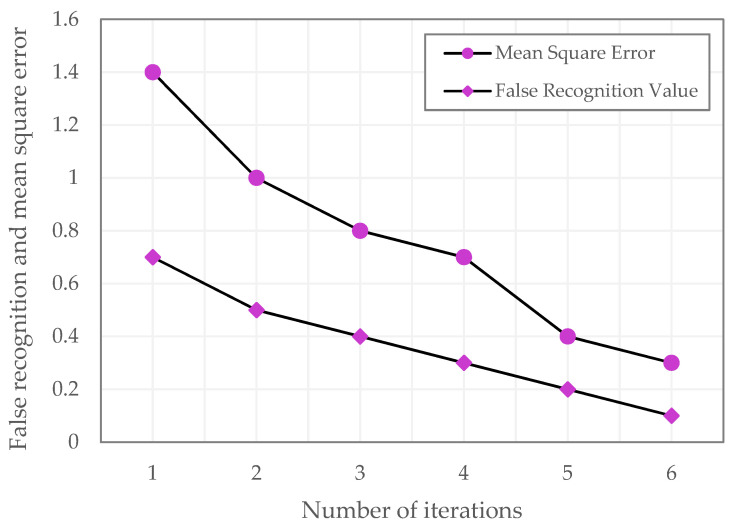
LPRNN training outcome.

We used two datasets of ultrasound breast images as the object, scaled the exploratory image to the respective ultrasound image in pixels, fed it into the LPRNN for iterative trials, and tracked the connection among both the mean square error and false detection accuracy of image local speckle noise identification as the total count of iterations changed. Figure 5, shows this to be the case. Table 1 illustrates the LPRNN training outcome in terms of both mean square error and false identification rates. Figure 5 depicts the graphical view of LPRNN training outcomes.

Figure 5, shows that the training MSE for the LPRNN using the proposed algorithm is consistently higher than the rate of incorrect identification. Both the mean square error and the false recognition rates decrease as the number of iterations increases. The mean square error and false recognition rate both fell to about 1.2% after 100 iterations. The fact that the mean square error and false recognition rates of image local speckle noise identification converge to small values shows that the LPRNN is superior for ultrasound image training. Table 2 illustrates the image noise removal state-of-art method.

**Figure 6:**
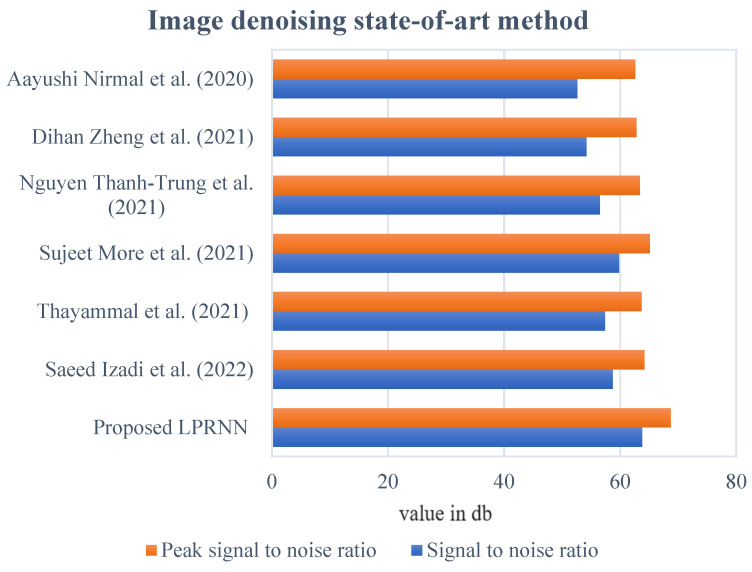
Graphical view of image noise removal different state-of-art-method [5,6,8,9,11,14].

As the experimental object, a sample of ultrasound breast images was chosen randomly from the two data sets, and local speckle noise destruction tests were carried out using the proposed technique to evaluate the signal-to-noise ratio (SNR) and peak SNR. In addition, the image noise-removal ultrasonic images had thresholds of 50 dB for anticipated SNR and 65 dB for peak ratio. Figure 6, shows that the SNR and peak SNR of the ultrasound images processed by the proposed method were both higher than the experiment thresholds of 60 dB and 65 dB after the local speckle noise was removed. This exemplifies the superior effectiveness of the proposed algorithm over state-of-the-art approaches in noise removal from breast ultrasound images. The proposed technique was tested on a dataset consisting of ultrasound breast images with a BPI threshold of 0.45, and the results were statistical analyses of the EPI data collected after suppression. 

**Figure 7:**
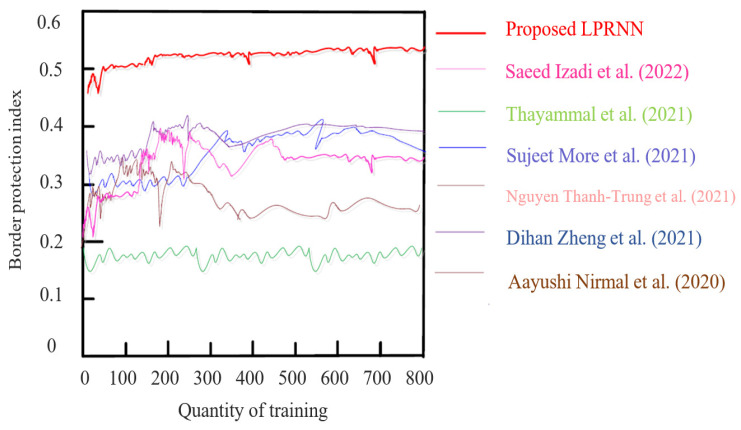
BPI after local speckle noise-removal [5,6,8,9,11,14].

**Figure 8:**
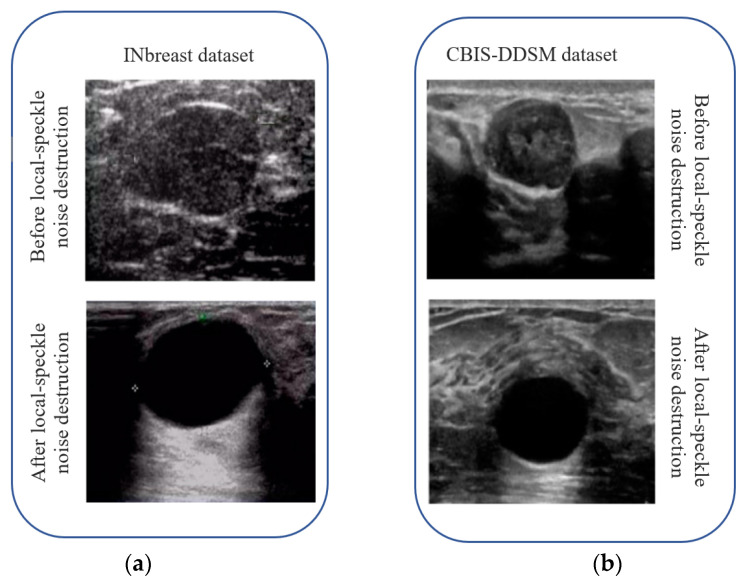
INbreast and CBIS-DDSM dataset image comparison (**a**) before and after the destruction; (**b**) before and after the local speckle destruction.

Figure 7 depicts the BPI range after local speckle noise destruction. Selecting an ultrasound breast image from the INbreast dataset and the CBIS-DDSM dataset allowed us to denoise the local speckle noise in the ultrasound breast image and examine the intuitive impact of the proposed method on the noise removal of ultrasound image local speckle noise. Figure 8 shows the difference in image quality before and after speckle noise reduction was applied. Figure 8, shows that local speckles were abundant in the initial ultrasound breast images taken from both datasets, leading to erroneous conclusions about the breast’s health and the potential for minor lesions to be missed.

**Figure 9:**
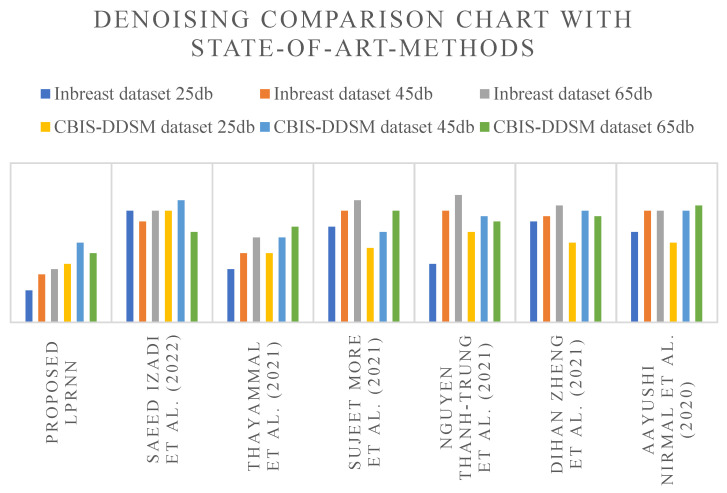
Graphical view of image noise removal comparison chart of proposed and state-of-art methods [5,6,8,9,11,14].

Contrarily, when local speckle noise is eradicated using the proposed approach, ultrasound image details are normal and clear, and the images are smooth and evenly distributed. In addition, following ultrasound image refining, the proposed technique has the effect of preserving edge information and making features more visible, allowing for more accurate monitoring of breast health. This demonstrates that the proposed approach successfully denoises ultrasound breast images by eliminating local speckle noise without losing any useful information. The ultrasound breast images in the INbreast dataset and the CBIS-DDSM dataset were each subjected to white Gaussian noise at levels of 25 dB, 45 dB, and 65 dB, and the algorithms’ denoising outcomes were compared based on PSNR and the amount of time it took to process the images. Table 3 illustrates the outcomes of these experiments. Abbreviation describes the list of shortforms and their respective abbreviations. Figure 9 depicts the noise removal comparison chart with a graphical view of proposed and state-of-art methods. Table 3 shows that when white Gaussian noise is added to the INbreast and DDSM datasets in varying levels, the proposed approach has lower noise overall and a much smaller peak noise compared to the other algorithms. The proposed method has reduced noise sensitivity and better local speckle noise removal. 

## 5. Discussion

As can be observed in Figure 6, the BPI values of the denoised ultrasound images processed using the proposed technique were all high and far above the experimental threshold of 0.45. For denoised ultrasound images, the methods of Saeed Izadi et al. (2022) [6], Thayammal et al. (2021) [5], and Sujeet More et al. (2021) [8] achieve BPI values of 0.40–0.45. For ultrasound images, the Nguyen Thanh-Trung et al. (2021) [11] method has a lower BPI, with a maximum value of just 0.33, while the Dihan Zheng et al. (2021) [14] algorithm consistently has a BPI below 0.2. This clearly demonstrates that the proposed algorithm does not cause a loss of edge information while local speckle noise is suppressed, resulting in good visualization of ultrasound breast images.

## 6. Conclusions

Clinical objectivity and manipulation are greatly enhanced when ultrasound breast images are processed and used using a LPRNN. This work employs a LPRNN, an algorithm of a guided filter, and high-pass filtering of a spatial method to destroy ultrasound image local speckle noise. In experiments, the proposed technique suppresses local speckle noise in ultrasound breast images, retains edge information, and makes image features apparent, laying the groundwork for intelligent ultrasound image processing and application. This proposed work may not examine the various types of noise and other features of ultrasound image characteristics. We guarantee that our future research will look into additional details about various image noises and breast image characteristics in order to improve the comprehension and clinical use of ultrasound images.

## Figures and Tables

**Table 1:** LPRNN training results, both mean square error and false recognition rate.

No. of Iterations	Mean Square Error	False Recognition Value
1	1.4	0.7
2	1	0.5
3	0.8	0.4
4	0.7	0.3
5	0.4	0.2
6	0.3	0.1

**Table 2:** Image noise removal state-of-art method.

Methods	Signal-to-Noise Ratio	Peak Signal-to-Noise Ratio
	Value in dB	
Proposed LPRNN	63.8	68.7
Saeed Izadi et al. (2022) [6]	58.7	64.2
Thayammal et al. (2021) [5]	57.4	63.7
Sujeet More et al. (2021) [8]	59.8	65.1
Nguyen Thanh-Trung et al. (2021) [11]	56.5	63.4
Dihan Zheng et al. (2021) [14]	54.2	62.8
Aayushi Nirmal et al. (2020) [9]	52.6	62.6

**Table 3:** Image noise removal comparison chart of proposed and state-of-art methods.

Methods	INbreast Dataset			CBIS-DDSM Dataset		
	25 db	45 db	65 db	25 db	45 db	65 db
Proposed LPRNN	6	9	10	11	15	13
Saeed Izadi et al. (2022) [6]	21	19	21	21	23	17
Thayammal et al. (2021) [5]	10	13	16	13	16	18
Sujeet More et al. (2021) [8]	18	21	23	14	17	21
Nguyen Thanh-Trung et al. (2021) [11]	11	21	24	17	20	19
Dihan Zheng et al. (2021) [14]	19	20	22	15	21	20
Aayushi Nirmal et al. (2020) [9]	17	21	21	15	21	22

## Data Availability

Not applicable.

## Abbreviation

**Short Form**	**Abbreviation**
LPRNN	Logical-Pool Recurrent Neural Network
SNR	Signal-to-Noise Ratio
PSNR	Peak Signal-to-Noise Ratio
DCNN	Deep Convolutional Neural Network
IR	InfraRed
3D	Three Dimensional
KSVD	K-singular Value Decomposition
SARN	Spatial-wise Attention Residual network
CNN	Convolutional Neural Network
JPEG	Joint Photographic Experts Group
MRI	Magnetic Resonance Imaging
ISO	International Organization for Standardization
CBIS-DDSM	Curated Breast Imaging Subset-Digital Database for Screening Mammography
XML	Extensible Markup Language
SAR	Specific Absorption Rate
MSE	Mean Square Error
BPI	Border Protection Index
